# A Naturally Occurring Plant Cysteine Protease Possesses Remarkable Toxicity against Insect Pests and Synergizes *Bacillus thuringiensis* Toxin

**DOI:** 10.1371/journal.pone.0001786

**Published:** 2008-03-12

**Authors:** Srinidi Mohan, Peter W. K. Ma, W. Paul Williams, Dawn S. Luthe

**Affiliations:** 1 Department of Entomology, Mississippi State, Starkville, Mississippi, United States of America; 2 USDA-ARS Corn Host Plant Resistance Laboratory, Mississippi State, Starkville, Mississippi, United States of America; 3 Department of Crop and Soil Sciences, The Pennsylvania State University, University Park, Pennsylvania, United States of America; Max Planck Institute for Chemical Ecology, Germany

## Abstract

When caterpillars feed on maize (*Zea maize* L.) lines with native resistance to several Lepidopteran pests, a defensive cysteine protease, Mir1-CP, rapidly accumulates at the wound site. Mir1-CP has been shown to inhibit caterpillar growth *in vivo* by attacking and permeabilizing the insect's peritrophic matrix (PM), a structure that surrounds the food bolus, assists in digestion and protects the midgut from microbes and toxins. PM permeabilization weakens the caterpillar defenses by facilitating the movement of other insecticidal proteins in the diet to the midgut microvilli and thereby enhancing their toxicity. To directly determine the toxicity of Mir1-CP, the purified recombinant enzyme was directly tested against four economically significant Lepidopteran pests in bioassays. Mir1-CP LC_50_ values were 1.8, 3.6, 0.6, and 8.0 ppm for corn earworm, tobacco budworm, fall armyworm and southwestern corn borer, respectively. These values were the same order of magnitude as those determined for the *Bacillus thuringiensis* toxin Bt-CryIIA. In addition to being directly toxic to the larvae, 60 ppb Mir1-CP synergized sublethal concentrations of Bt-CryIIA in all four species. Permeabilization of the PM by Mir1-CP probably provides ready access to Bt-binding sites on the midgut microvilli and increases its activity. Consequently, Mir1-CP could be used for controlling caterpillar pests in maize using non-transgenic approaches and potentially could be used in other crops either singly or in combination with Bt-toxins.

## Introduction

Since the beginning of agriculture, crops have been beleaguered by insect pests. Even now, insect herbivory is responsible for 10 to 20% of major crop losses worldwide [1], [2]. Traditionally, chemical pesticides have been used to control insect damage and at one time it was estimated that approximately 40% of chemical pesticide expenditures have been for controlling Lepidopteran pests alone [3]. Although the use of “hard” chemical insecticides has provided some control, their toxicity to non-target animals and humans poses a serious negative effect on the environment [1], [2], [4], [5]. In the mid-1990s, farmers planted the first transgenic crops expressing the Bt-gene (*Bt*) that encodes the Δ-endotoxin of *Bacillus thuringiensis* to control Lepidopteran pests [2], [4]–[6]. By 2006, approximately 30 million hectares of crops expressing Bt-gene were planted globally [7]. Genetically modified, insect-resistant crops, especially cotton and maize, have been a boon to farmers in both the developed and developing world. Because the toxicity of the Δ-endotoxins (Cry-toxins) is generally specific for certain insects, transgenic crops expressing the Cry-toxins have proved to be an “environmentally friendly” method of pest control.

While Bt-crops have been widely accepted by the agricultural community, there are nagging concerns about the durability of the technology [8]–[11]. One concern about the widespread use of transgenic crops expressing a single Bt gene is that the target insect populations will develop resistance to the toxin thus reducing its long-term effectiveness [8]–[11]. It has been demonstrated that field populations of the diamondback moth (*Plutella xylostella)* have developed resistance to Bt-toxin [12], [13], but resistance against transgenic Bt-toxin expressing plants among other Lepidopteran larvae has yet to be seen in crops [10]. However, recent studies indicate that there are differences in sensitivity to Bt-CryIAc and Bt-CryIIAb2 among field and laboratory colonies of corn earworm and tobacco budworm [14], [15] and European corn borer [16]. It has been suggested that the stacking or pyramiding of genes with different modes of action can significantly increase the number of generations needed for the target insects to become resistant [8], [17], [18], [19].

A series of steps are required for the Bt-toxins to exert their effect on Lepidopteran larvae. First, larvae must ingest the Bt-protoxin, which is converted to its active form by proteases in the alkaline midgut environment [20], [21]. The toxin then must pass through the peritrophic matrix (PM) and bind to specific midgut receptors on the brush border membrane with very high affinity [22], [23]. This is followed by irreversible insertion into the membrane [24], which then forms lytic pores resulting in cell lysis, cessation of feeding and larval death [25]. Experiments using laboratory populations of Lepidoptera suggests that there are three potential resistance mechanisms [26]. The most predominant is the alteration of Bt-toxin binding site on the midgut receptors, which reduces the toxin's ability to bind to the cells [27]. This may be due to decreased binding affinity or a reduction in the number of binding sites. Other possible resistance mechanisms include alterations in the proteolytic processing of the Bt-protoxin and rapid regeneration of the damaged midgut epithelium [27]. To counteract the development of resistance in Lepidopteran field populations, it would be beneficial to have additional resistance technologies with varying modes of action available.

Maize lines with inherent resistance to armyworm (*Spodoptera frugiperda*) and other Lepidopteran larvae have been developed from exotic germplasm from Antiqua using conventional plant breeding [28], [29]. Within one hour, these lines accumulate maize insect resistance cysteine protease (Mir1-CP) in the whorl in response to Lepidopteran larval feeding [30]. This rapid accumulation occurs because Mir1-CP is located in the maize vascular tissues, which allows it to be rapidly mobilized in response to herbivory [31]. When fall armyworm larvae were fed on transgenic maize callus tissue ectopically producing Mir1-CP, their growth was inhibited approximately 70% [30]. Subsequent scanning electron microscopy (SEM) studies indicated that the peritrophic matrix (PM), of larvae feeding on the transgenic callus was severely damaged and contained numerous holes and fissures [32]. The PM is an extracellular matrix of chitin, glycoproteins and proteoglycans that surrounds the food bolus and protects the midgut epithelium from damage and assists in nutrient uptake and cycling [33]. *In vitro* studies using purified recombinant Mir1-CP indicated that it completely permeabilizes the PM by digesting PM proteins [34]. These studies also indicated that Mir1-CP was most effective on Lepidopteran larvae belonging to the Noctuidae family, the largest and most economically important familiy of Lepidopterans [34].

Mir1-CP is a papain-like cysteine protease that interestingly has amino acid sequence similarity to cysteine proteases from several baculoviruses that infect Lepidopteran larvae such as *A. californica* and *Spodoptera exigua* nucleopolyhedrovirus [35], [36]. Another baculovirus metalloprotease, enhancin, attacks the PM of *Trichoplusia ni* and specifically degrades the integral PM protein, Insect Intestinal Mucin (IIM) [37], [38]. However, Mir1-CP is the only plant defensive cysteine protease that has been shown to directly damage the Lepidopteran PM. Because Mir1-CP acts catalytically, it should have much greater efficacy against larvae than other plant defense proteins such as protease inhibitors and lectins [39].

The idea of stacking other resistance genes with Bt is not new, but to the best of our knowledge there have been no reports of incorporating Bt resistance with naturally occurring plant defense proteins. Mir1-CP and Bt-toxins have different, but potentially complementary toxicity mechanisms. Mir1-CP functions by permeabilizing the PM, whereas Bt-toxins bind the intestinal microvilli. Therefore, this study was undertaken to directly measure the toxicity of Mir1-CP on several different types Lepidopteran larvae and to determine if it acts synergistically with the Bt-toxin CryIIA in bioassays. Mir1-CP and Bt-CryIIA were tested on corn earworm (*Helicoverpa zea, Noctuidae*) and tobacco budworm (*Heliothis virescens, Noctuidae*), fall armyworm (*Noctuidae*) and southwestern corn borer (*Diatraea grandiosella, Crambidae*). These insects are economically important pests of many crops that are targets of Bt-producing transgenic plants. The results indicated that Mir1-CP had LC_50_ values on par with those of Bt-CryIIA and that the remarkably low concentration of 60 ppb could dramatically synergize sublethal concentrations of the Bt-toxin.

## Results

### LC_50_ values

Table 1 shows the LC_50_ values for corn earworm, tobacco budworm, fall armyworm, and southwestern corn borer fed either purified Mir1-CP or the processed form of the Bt-toxin, CryIIA. The noctuids, corn earworm, tobacco budworm and fall armyworm were the most sensitive to Mir1-CP with LC_50_ values ranging from 0.6 to 3.6 ppm. The crambid, southwestern corn borer was less sensitive with a LC_50_ of 8.0 ppm. The LC_50_ values for CryIIA were similar among all of the larvae tested and ranged from 0.9 to 1.5 ppm. These values indicate that purified recombinant Mir1-CP is effective at low concentrations and has LC_50_ values that are the same order of magnitude as those of CryIIA.

**Table 1:** Probit analysis of LC_50_ values for several Lepidopteran species fed Mir1-CP and Bt-CryIIA. LC_50_ values are in ppm and numbers in parenthesis represent the 95% confidence interval.

Insects	Mir1-CP			Bt-CryIIA		
	*LC_50_	Slope (±SE)	R^2^	*LC_50_	Slope (±SE)	R^2^
Corn earworm	1.8 (1.2–3.2)	1.202 (0.14)	0.98	0.9 (0.5–1.7)	1.084 (0.18)	0.98
Tobacco budworm	3.6 (3.0–4.5)	1.461 (0.08)	0.93	1.2 (0.8–1.7)	1.022 (0.13)	0.96
Fall armyworm	0.6 (0.3–1.1)	0.722 (0.18)	0.97	1.1 (0.8–1.3)	0.913 (0.06)	0.94
Southwestern corn borer	8.0 (6.9–10.1)	1.250 (0.40)	0.96	1.5 (1.0–3.5)	0.965 (0.14)	0.97

### Combined effects of Mir1-CP and CryIIA on larval growth and mortality

**Figure 1:**
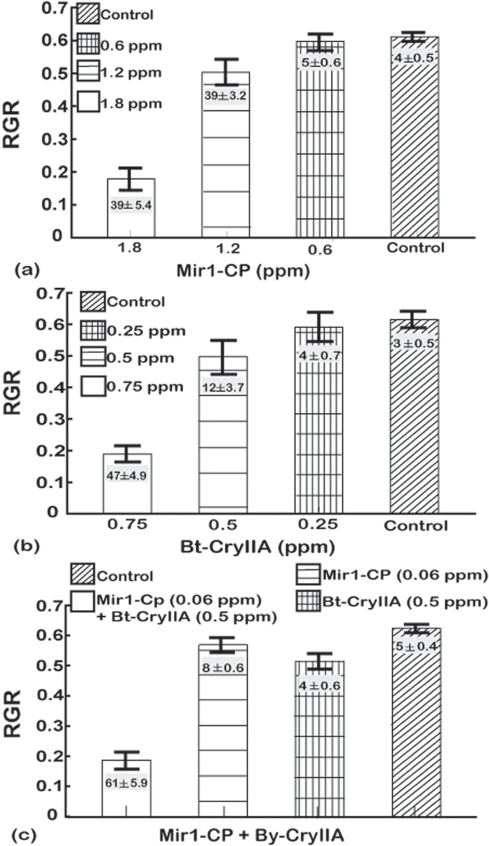
Dose response analysis for corn earworm larvae fed (a) Mir1-CP, (b) Bt-CryIIA, and (c) sub-lethal doses of Bt-CryIIA and Mir1-CP. The y-axis is the relative growth rate (RGR measured as Δmg/(mg_avg_d) and vertical bars indicate one standard deviation. The least significant difference (LSD) at the 0.05 confidence level for the RGR values were as follows: Mir1-CP = 0.25, Bt-CryIIA = 0.3, sub-lethal doses of Bt-CryIIA and Mir1-CP = 0.42. Numbers (mean±SD) in the histogram bars are the average percentage mortality for each treatment. The least significant difference (LSD) at the 0.05 confidence level for mortality were as follows: Mir1-CP – 7.4, Bt-CryIIA – 9.5, sub-lethal doses of Bt-CryIIA and Mir1-CP combined – 15.94. Results are based on four independent bioassays. Concentrations are given as parts per million (ppm).

A series of experiments were done for each Lepidopteran species to determine concentrations of Mir1-CP and CryIIA that slightly inhibited larval growth and resulted in limited mortality. When larvae of the four species were reared on artificial diet alone, they all had similar relative growth rates (RGR) of 0.60±0.06 and mortality ranged from 2 to 8% (Fig. 1 to [Fig pone-0001786-g002] [Fig pone-0001786-g003] 4).

**Figure 2 pone-0001786-g002:**
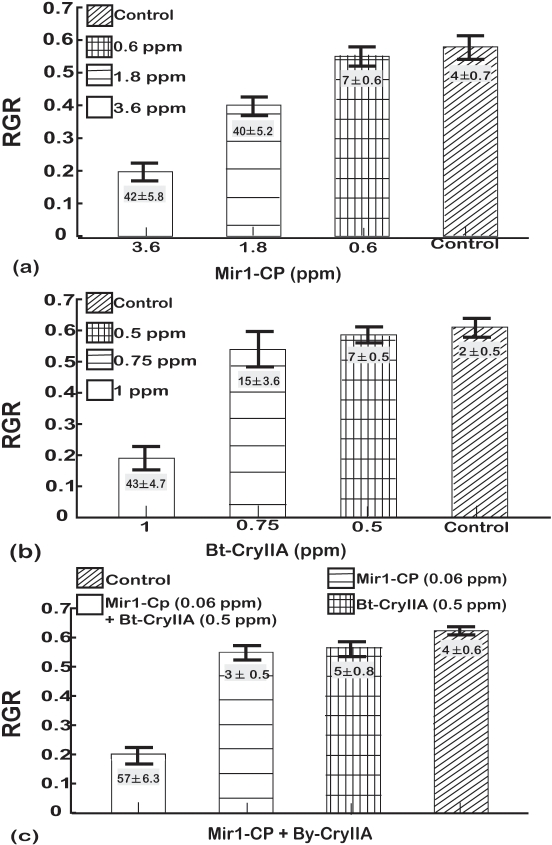
Dose response analysis for tobacco budworm larvae fed (a) Mir1-CP, (b) Bt-CryIIA, and (c) sub-lethal doses of Bt-CryIIA and Mir1-CP. The y-axis is the relative growth rate (RGR measured as Δmg/(mg_avg_d) and vertical bars indicate one standard deviation. The least significant difference (LSD) at the 0.05 confidence level for the RGR values were as follows: Mir1-CP – 0.31, Bt-CryIIA – 0.44, sub-lethal doses of Bt-CryIIA and Mir1-CP combined – 0.57. Numbers (mean±SD) in the histogram bars are the average percentage mortality for each treatment. The least significant difference (LSD) at the 0.05 confidence level for mortality were as follows: Mir1-CP – 8.5, Bt-CryIIA – 7.4, sub-lethal doses of Bt-CryIIA and Mir1-CP combined – 15.83. Results are based on four independent bioassays. Concentrations are given as parts per million (ppm).

**Figure 3 pone-0001786-g003:**
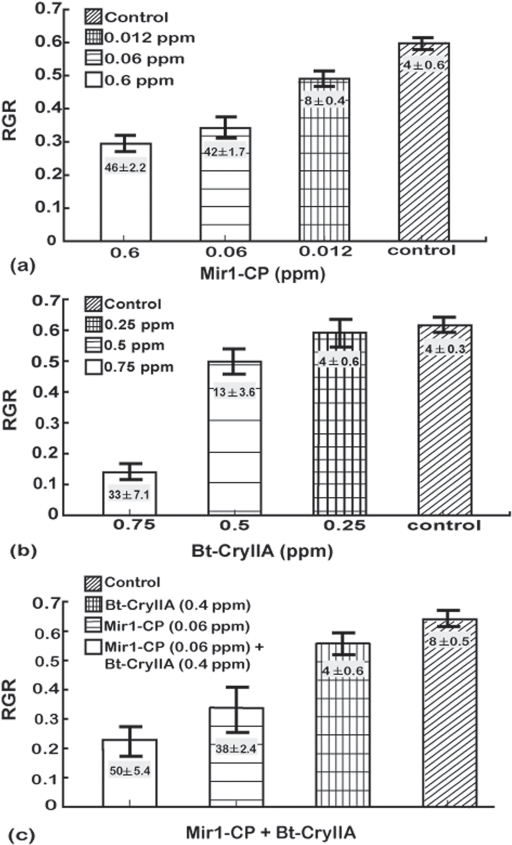
Dose response analysis for fall armyworm larvae fed (a) Mir1-CP, (b) Bt-CryIIA, and (c) sub-lethal doses of Bt-CryIIA and Mir1-CP. The y-axis is the relative growth rate (RGR measured as Δmg/(mg_avg_d) and vertical bars indicate one standard deviation. The least significant difference (LSD) at the 0.05 confidence level for the RGR values were as follows: Mir1-CP – 0.14, Bt-CryIIA – 0.37, sub-lethal doses of Bt-CryIIA and Mir1-CP, combined – 0.46. Numbers (mean±SD) in the histogram bars are the average percentage mortality for each treatment. The least significant difference (LSD) at the 0.05 confidence level for mortality were as follows: Mir1-CP – 6.71, Bt-CryIIA – 8.42, sub-lethal doses of Bt-CryIIA and Mir1-CP, combined – 10.01. Results are based on four independent bioassays. Concentrations are given as parts per million (ppm).

**Figure 4:**
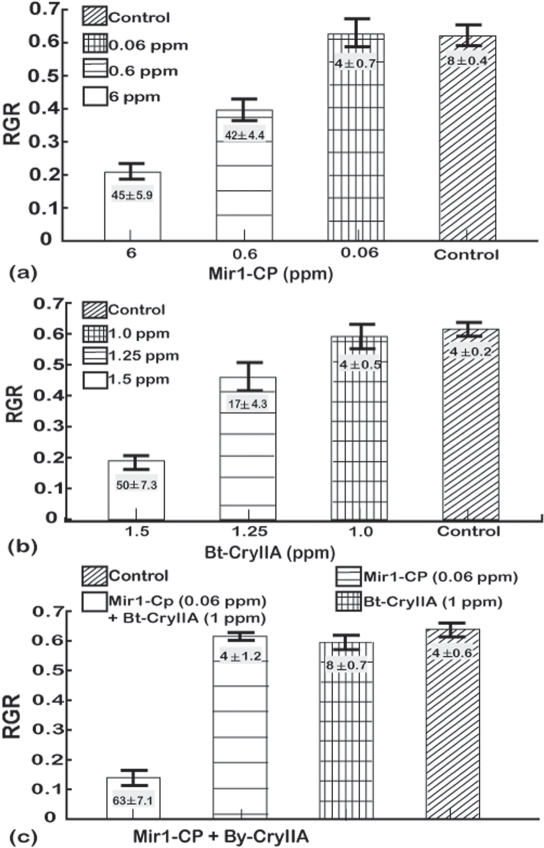
Dose response analysis for southwestern corn borer larvae fed (a) Mir1-CP, (b) Bt-CryIIA, and (c) sub-lethal doses of Bt-CryIIA and Mir1-CP. The y-axis is the relative growth rate (RGR measured as Δmg/(mg_avg_d) and vertical bars indicate one standard deviation. The least significant difference (LSD) at the 0.05 confidence level for the RGR values were as follows: Mir1-CP – 0.15, Bt-CryIIA – 0.23, sub-lethal doses of Bt-CryIIA and Mir1-CP combinded – 0.38. Numbers (mean±SD) in the histogram bars are the average percentage mortality for each treatment. The least significant difference (LSD) at the 0.05 confidence level for mortality were as follows: Mir1-CP – 8.97, Bt-CryIIA – 9.24, sub-lethal doses of Bt-CryIIA and Mir1-CP combined – 16.88. Results are based on four independent bioassays. Concentrations are given as parts per million (ppm).

For corn earworm, 1.80 ppm of Mir1-CP reduced the RGR to 0.18±0.03 and resulted in 39±5.4% mortality (Fig. 1a). Bt-CryIIA (0.75 ppm) reduced the RGR to 0.19±0.02 and the mortality was 47±4.9% (Fig. 1b). Doses of either 0.60 ppm Mir1-CP or 0.25 ppm Bt-CryIIA did not significantly decrease the RGR or percentage mortality relative to the control (Fig. 1a, b). When corn earworm larvae were fed a combination of Mir1-CP and Bt-CryIIA at concentrations (0.06 ppm and 0.5 ppm, respectively) that had little or no effect on the larvae when used singly, the RGR dramatically dropped to 0.20±0.01, and the percentage mortality increased to 61±5.9% (Fig. 1c, Table 2).

**Table 2:** Relative growth rate (RGR) and percentage mortality of Lepidopteran larvae fed on Mir1-CP, Bt-CryIIA or Mir1-CP and Bt-CryIIA in combination.

	Mir1-CP (ppm)	(±SE)	Bt-CryIIA (ppm)	(±SE)	Combination	(±SE)	P value
Corn earworm							
Dose	0.06		0.5		0.06+0.5		
Mortality (%)	8	0.60	4	0.60	61	5.9	0.0315
RGR	0.574	0.04	0.514	0.02	0.197	0.03	0.0370
Tobacco budworm							
Dose	0.06		0.5		0.06+0.5		
Mortality (%)	3	0.50	5	0.80	57	6.3	0.0034
RGR	0.562	0.03	0.574	0.02	0.201	0.02	0.0390
Fall armyworm							
Dose	0.06		0.4		0.06+0.4		
Mortality (%)	38	2.4	4	0.60	50	5.4	0.0123
RGR	0.333	0.08	0.568	0.05	0.22	0.03	0.0330
Southwestern corn borer							
Dose	0.06		1		0.06+1		
Mortality (%)	4	1.2	8	0.70	63	7.1	0.0392
RGR	0.609	0.01	0.596	0.02	0.132	0.02	0.0420

RGR = Δmg/(mg_avg_d); SE = standard error. P values indicate that % mortality and RGR values significantly differ from additivity and show synergy at the 95% confidence level.

Tobacco budworm larvae required 3.6 ppm of Mir1-CP to decrease the RGR to 0.21±0.02 (Fig. 2a) and increase the mortality to 42±5.8%. One ppm of Bt-CryIIA reduced the RGR to 0.20±0.02 (Fig. 2b) and increased the mortality to 43±4.7%. The lower doses of 0.6 ppm Mir1-CP and 0.5 ppm of Bt-CryIIA did not significantly decrease the RGR or percentage mortality (Fig. 2a, b) relative to the control. However, when 0.06 ppm Mir1-CP and 0.5 ppm of Bt-CryIIA were combined, the RGR was far lower (0.19±0.03) and the mortality increased to 57±6.3% (Fig. 2c, Table 2).

The fall armyworm RGR decreased to 0.31±0.04 at 0.60 ppm Mir1-CP and was 0.18±0.03 with 0.75 ppm Bt-CryIIA (Fig. 3a, b). Mortality was 46±2.2% and 33±7.1% at the highest concentrations of Mir1-CP and Bt-CryIIA tested, respectively. When fall armyworm larvae were treated with 0.06 ppm of Mir1-CP, the RGR was 0.33±0.04 and mortality was 42±1.7% (Fig. 1a). Larvae reared on 0.25 ppm Bt-CryIIA had a RGR of 0.64±0.04, which was not significantly different from the control and mortality was the same as the control (4%) (Fig. 3b). At a concentration of 0.5 ppm Bt-CryIIA, the RGR decreased to 0.50±0.06 and mortality was 13±3.6% (Fig. 1b). Therefore, the concentrations of 0.06 ppm Mir1-CP and 0.4 ppm Bt-CryIIA were selected to test the effect of the components in combination (Fig. 3c). When these two doses of Mir1-CP and Bt-CryIIA were combined, the RGR value was reduced to 0.22±0.05 (Fig. 1c) and the percentage mortality increased to 50±5.4%, which was somewhat higher than those of the individual treatments (Table 2).

The southwestern corn borer was more recalcitrant to both Mir1-CP and Bt-CryIIA. Six ppm of Mir1-CP were required to reduce the RGR to 0.21±0.02 and increase the mortality to 45±5.9% (Fig. 4a). A concentration of 1.5 ppm Bt-CryIIA was needed to reduce the RGR to 0.19±0.02 and increase the mortality to 50±7.3% (Fig. 4b). When used singly, the concentrations 0.06 ppm of Mir1-CP and 1 ppm Bt-CryIIA did not significantly change the RGR or mortality (4% for each Fig. 4c) relative to the control. In combination, however, the effect was much greater as the RGR was reduced to 0.13±0.03 and the mortality increased to 63±7.1% (Fig. 4c, Table 2).

The data for all four lepidopteran species indicates that 0.06 ppm of Mir1-CP can synergize sublethal concentrations of Bt-CryIIA resulting in significantly lower growth rates and higher mortality.

**Figure 5:**
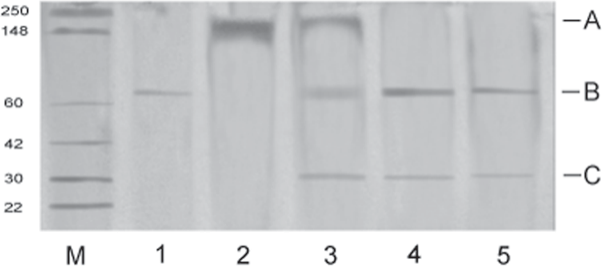
SDS-PAGE analysis of Bt-CryIIA protoxin and its processed form incubated with 0.06 ppm of Mir1-CP. Lane M – markers; lane 1 -processed Bt-CryIIA; lane 2 – unprocessed Bt-CryIIA protoxin; lane 3 - unprocessed Bt-CryIIA protoxin incubated with Mir1-CP for 8 hr; lane 4 – unprocessed Bt-CryIIA protoxin incubated with Mir1-CP for 24 hr; lane 5 – processed Bt-CryIIA toxin incubated with Mir1-CP for 8 hr. Numbers on the left margin refer to the molecular mass of the marker bands in kD. Arrow A indicates the position of the protoxin, arrow B indicates the position of the processed toxin and arrow C marks the position of Mir1-CP (molecular mass of 33 kD) added to the incubation.

We suggest there are two possible ways that Mir1-CP could synergize Bt-endotoxin. First, Mir1-CP, which permeabilizes the PM, likely facilitates Bt-CryIIA movement though the PM allowing greater access to the epithelial cells. Alternatively, Mir1-CP might process the Bt-CryIIA protoxin to the toxic form in the bioassay or the midgut. Because the processed form of the toxin was used in the bioassays, the second possibility is unlikely. Nevertheless, the ability of Mir1-CP to process the Bt-CryIIA protoxin to its mature form was tested by incubating the protoxin with Mir1-CP. SDS-PAGE analysis indicated that Mir1-CP partially cleaved the protoxin after 8 hours of incubation (Fig. 5, lane 3) and completely cleaved it after 24 hours (Fig. 5, lane 4). However, it did not affect the mobility of the processed form that was used in the bioassays (Fig. 5, lane 5). The low molecular weight band (Fig. 5, lanes 3, 4, and 5) is Mir1-CP. If Mir1-CP is capable of processing the protoxin in vivo, it might enhance its synergistic effects.

## Discussion

Our study demonstrates that Mir1-CP, a natural defense cysteine protease found in maize [29], [30], reduces the growth of Lepidopteran larvae when directly added to artificial diet and synergizes the effects of the Bt-toxin CryIIA. LC_50_ values for Mir1-CP ranged from 0.9 to 8.0 ppm and were in the same range as those of Bt-CryIIA. In fact, Mir-CP appears to be considerably more toxic than other plant-derived defense proteins. For example, protease inhibitors do not act catalytically and their effective concentrations range from 1 to 5% of the total protein in the diet [39]. There was a 10-fold range in sensitivity to Mir1-CP among the Lepidopteran species tested and Southwestern corn borer was the least sensitive to the protease. The LC_50_ values correlate well with *in vitro* permeability studies, which also indicated that PM in the Noctuidae were more sensitive to Mir1-CP degradation than those of the Crambidae [34]. Preliminary evidence suggests that that the Southwestern corn borer PM may contain one or more proteins that are resistant to Mir1-CP attack (Luthe and Mohan, unpublished data).

Both scanning electron microscopy (SEM) and *in vitro* permeability experiments indicated that the PM is the target of Mir1-CP [32], [34]. SEM indicated that there were many cracks and fissures in the PM of fall armyworm larvae that fed on plant material containing Mir1-CP [32]. The *in vitro* studies indicated that the PM could be completely permeabilized by Mir1-CP in a concentration dependent manner [34]. Therefore, it appears that Mir1-CP exerts its effect on these Lepidopterans by making holes in the PM. Damage to the PM results in the loss of the midgut protective barrier, disrupts nutrient cycling in the midgut and slows larval growth [33]. The Bt-endotoxins have a different toxicity mechanism than Mir1-CP and act by binding to receptors in the midgut cells and forming lytic pores [24], [40]. Because the processed form of BT-CryIIA was used in this study, Mir1-CP probably synergized the activity of Bt-CryIIA, by increasing PM permeability and facilitating toxin movement to the microvilli. Consequently, it is likely that Mir1-CP will also synergize other Bt-toxins including CryIAb and CryIAc, which are typically used in transgenic crops. Our data also indicate that Mir1-CP processes the protoxin to its mature form *in vitro*. If Mir1-CP were deployed in transgenic plants expressing the protoxin, it might have dual functions: processing the protoxin and perforating the PM.

This study shows that Mir1-CP is effective against Lepidopteran pests and may be one of the most toxic endogenous plant defense proteins known and that only 60 ppb of Mir1-CP are required to synergize the effects of Bt-CryIIA concentrations ranging from 0.40 to 1.0 ppm. The synergism between Mir1-CP and Bt-CryIIA suggests that stacking these two genes in transgenic plants could enhance the effectiveness of Bt-toxin, potentially reduce the development of insect resistance to this class of toxins, and ultimately result in more sustainable control of Lepidopteran pests.

### Experimental protocol

#### Chemicals and reagents

All chemicals and reagents were purchased from Fisher Biotech (Fairlawn, NJ). Purified 33-KDa Mir1-CP was prepared as previously described by Mohan et al. [31]. Processed 68-KDa Bt-CryIIA toxin and its unprocessed 143-KDa form were obtained from Monsanto Plant Science/Regulatory Sciences (St. Louis, MO).

The two Bt-CryIIA proteins were dissolved in 50 mM potassium phosphate, 50 mM sodium chloride, 1 mM EDTA, with at final pH of 10.8,

#### Insect rearing

Larvae of fall armyworm (*Spodoptera frugiperda*), southwestern corn borer (*Diatraea grandiosella*), corn earworm (*Helicoverpa zea*) and tobacco budworm (*Heliothis virescens)* were obtained from laboratory colonies maintained by the USDA-ARS Corn Host Plant Resistance Research Unit at Mississippi State University. They were reared on artificial diet [31] under a photoperiod of 16∶8 at 26.6°C.

#### Insect feeding bioassays

Dose response bioassays were conducted using second instar larvae of each of the Lepidopteran species. Artificial diet (approximately 250 µl) was placed in 96 well plates and the surface was covered with 50 µl solutions containing dilutions of either recombinant Mir1-CP, processed Bt-CryIIA or a combination of both. Untreated wells served as controls. The plates were air dried for 3–4 h and covered with Saran Wrap®. Prior to the bioassay, larvae were weighed and placed in individual wells and the plates were covered with Breath Easy™ (USA Scientific Inc, FL, USA). Each bioassay treatment involved 24 larvae and was performed with three replicates. Larvae were incubated at 30°C/48 h in 16∶8 photoperiod before final larval weight was measured. Previous experiments indicated that purified Mir1-CP lost only 20% of its activity after incubation at 30°C for 7 days (Pechan and Luthe, unpublished data). Consequently, Mir1-CP should retain a major portion of activity during the 48 hr bioassay. Each bioassay treatment condition was repeated independently three to five times. The larval relative growth rate (RGR) [41] was calculated using the formula RGR = ((final larval weight)-(initial larval weight))/((average larval weight) x time) and is reported as Δmg/(mg_avg_ d).

Experiments were conducted using a randomized complete block design with a factorial structure of Mir1-CP and Bt-CryIIA and statistical analyses were done using SAS® software (Cary, NC). Statistically significant departures from additivity were used to determine if there was a synergistic interaction between Mir1-CP and Bt-CryIIA for both RGR and the percentage mortality.

#### Toxicity analysis (LC_50_)

Dose response analysis was conducted to determine the LC_50_ for each of the Lepidopteran species. At least 24 larvae of each Lepidopteran species were tested against each concentration of either Mir1-CP or Bt-CryIIA toxin. Larvae were incubated at 30°C/48 h in 16∶8 photoperiod. Mortality was recorded after 24 h and each experimental condition was replicated at least four times. The LC_50_ was calculated using PROC PROBIT (SAS Institute 1998) [42]. Variability was examined using a one-way analysis of variance (ANOVA) (PROC ANOVA, SAS Institute 1998) [42]. Confidence intervals (CIs) were calculated using the Robertson and Preisler (1992) [43] methods as explained by Ali et al., (2006) [15]. The Mir1-CP concentrations tested, regression equation and coefficient (R^2^) for each species are given in parentheses following the insect name: corn earworm (1, 2, 3, 4, ppm; y = 2.4269x - 7.9283; R^2^ = 0.9869), tobacco budworm (1.2, 2.4, 3.6, 4.8 ppm; y = 1.3305x - 4.7327; R^2^ = 0.9349), fall armyworm (0.3, 0.6, 0.9, 1.2 ppm; y = 2.2104x - 6.1873; R^2^ = 0.9796), southwestern corn borer (3, 6, 9, 12 ppm; y = 0.4890x - 1.7891; R^2^ = 0.9605). The concentrations Bt-CryIIA concentrations tested, regression equation and coefficient (R^2^) for each species are given in parentheses following the insect name: corn earworm (0.75, 1.5, 2.1 ppm; y = 2.666 x - 7.8445; R^2^ = 0.9849), tobacco budworm (0.75, 1.5, 2.1 ppm; y = 3.1959x – 9.7953; R^2^ = 0.9658), fall armyworm (0.5, 1.0, 1.5 ppm; y = 2.2021x – 6.6875; R^2^ = 0.9435), southwestern corn borer (1.0, 2.0, 3.0 ppm; 7.8146x -24.847; R^2^ = 0.9759).

#### Processing of Bt-CryIIA protoxin

To determine if MIr1-CP could cleave the Bt-CryIIA protoxin, 12 µg of either the protoxin or processed Bt-CryII were incubated for 8 and 24 hr with 3 µg of Mir1-CP in a total volume of 15 µl water at room temperature. The sample was diluted (1∶1) with sample buffer [42] and boiled for 5 min. Samples containing 7.5 µg of protein were then analyzed by SDS-PAGE [44]. The gel was stained with colloidal Coomassie Blue as described by Neuhoff et al. [45]. Multimark™ Multi-colored protein ladder (Novex, CA) was used to determine the molecular weight of proteins in SDS-PAGE.
